# Inspection of intraductal papillary mucinous neoplasm via the papilla using a novel slim pancreatoscope under balloon enteroscopy

**DOI:** 10.1055/a-2239-2060

**Published:** 2024-01-30

**Authors:** Yuki Tanisaka, Masafumi Mizuide, Akashi Fujita, Takahiro Shin, Kei Sugimoto, Ryuhei Jinushi, Shomei Ryozawa

**Affiliations:** 1 183786 Department of Gastroenterology, Saitama Medical University International Medical Center, Hidaka, Japan

Peroral pancreatoscopy (POPS) is useful for the direct visualization of intraductal lesions in the pancreatic duct 1 2 3 . However, POPS in patients with Roux-en-Y anastomosis via the papilla under balloon enteroscopy is difficult because pancreatoscopes are approximately 10 Fr in diameter and cannot pass through the forceps channel of the balloon enteroscope. We report a successful inspection of an intraductal papillary mucinous neoplasm (IPMN) using a novel slim pancreatoscope under balloon enteroscopy in a patient with Roux-en-Y gastrectomy.

A 74-year-old man had undergone total gastrectomy with Roux-en-Y for gastric cancer 4 years earlier. On referral to our facility, computed tomography and magnetic resonance imaging revealed pancreatic duct dilation and a pancreatic cyst in the tail region ( Fig. 1 ). Endoscopic ultrasonography revealed pancreatic duct dilation and a pancreatic cyst with a suspected mural nodule connected with the main pancreatic duct ( Fig. 2 ). Therefore, endoscopic retrograde cholangiopancreatography (ERCP) was performed using a short-type single-balloon enteroscope (SIF-H290; Olympus, Tokyo, Japan) with a working length of 152 cm and a working channel diameter of 3.2 mm 4 5 . Additionally, POPS was performed using a slim pancreatoscope (DRES Slim Scope; Japan Lifeline, Tokyo, Japan) with a length of 195 cm and a diameter of 2.6 mm ( Fig. 3 , Video 1 ). Endoscopic findings revealed mucus discharge from the papilla ( Fig. 4 **a** ). Pancreatography revealed defects in the pancreatic tail ( Fig. 4 **b** ). Subsequently, POPS was performed using a slim pancreatoscope. A villous, protruding lesion was observed in the tail of the pancreatic duct, whereas no lesions were observed in the head and body of the pancreatic duct ( Fig. 5 ). Finally, we diagnosed the patient with IPMN with mural nodules in the tail of the pancreatic duct.

**Fig. 1:**
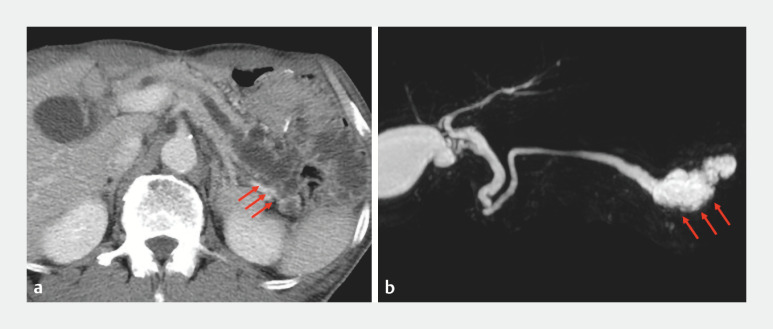
Findings revealing pancreatic duct dilation and pancreatic cyst in the tail region (red arrow). **a** Computed tomography. **b** Magnetic resonance imaging.

**Fig. 2:**
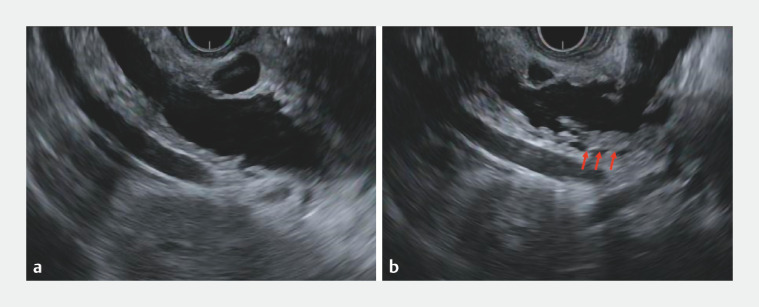
Endoscopic ultrasonography revealing pancreatic duct dilation and pancreatic cyst with a suspected mural nodule connected with the main pancreatic duct (red arrow).

**Fig. 3:**
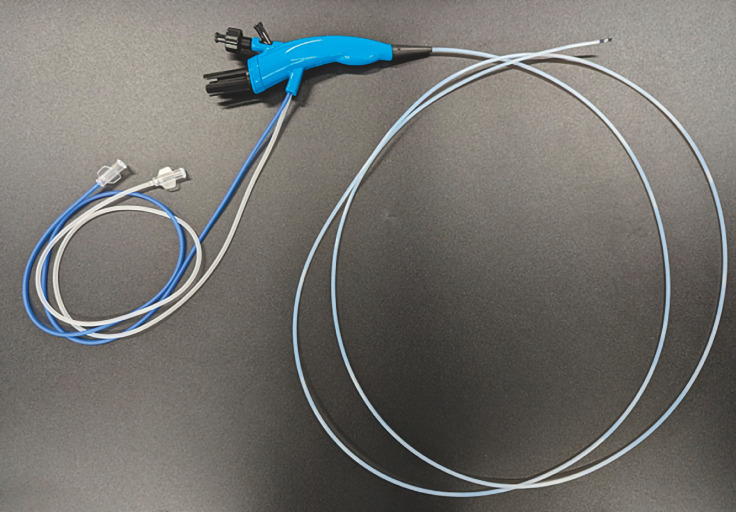
A slim pancreatoscope with a length of 195 cm and a diameter of 2.6 mm.

Successful inspection of intraductal papillary mucinous neoplasm via the papilla using a novel slim pancreatoscope under balloon enteroscopy in a patient with Roux-en-Y gastrectomy.Video 1

**Fig. 4:**
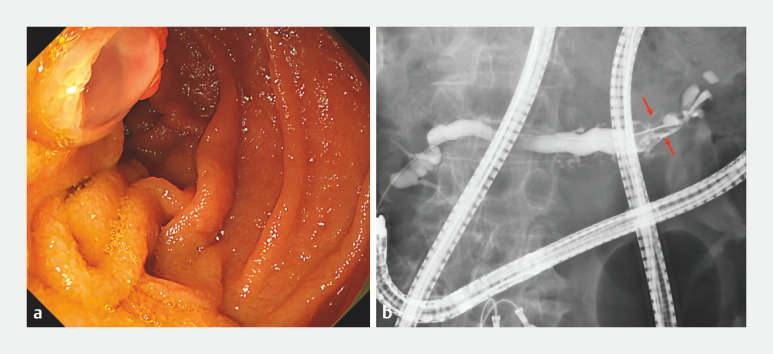
Endoscopic and pancreatography findings. **a** Endoscopic findings showing mucus discharge from the papilla. **b** Pancreatography revealing defects in the pancreatic tail (red arrow).

**Fig. 5:**
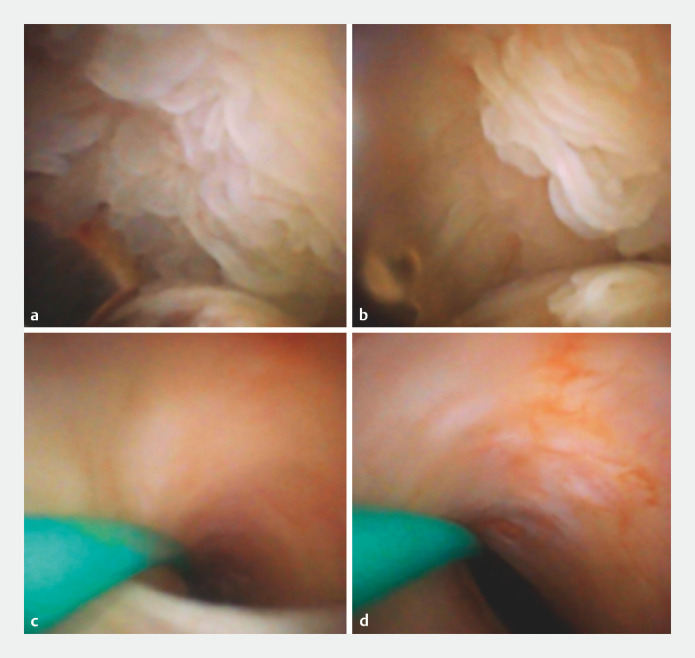
Peroral pancreatoscopy findings. **a,b** A villous, protruding lesion in the tail of the pancreatic duct. **c** No lesions were observed in the head of the pancreatic duct. **d** No lesions were observed in the body of the pancreatic duct.

Although POPS via the papilla is considered difficult in patients with Roux-en-Y anastomosis under balloon enteroscopy, this novel slim pancreatoscope makes it possible, potentially improving the diagnostic yield in such patients.

Endoscopy_UCTN_Code_TTT_1AR_2AI

## Notes

Endoscopy E-Videos https://eref.thieme.de/e-videos
*E-Videos* is an open access online section of the journal *Endoscopy* , reporting on interesting cases and new techniques in gastroenterological endoscopy. All papers include a high-quality video and are published with a Creative Commons CC-BY license. Endoscopy E-Videos qualify for HINARI discounts and waivers and eligibility is automatically checked during the submission process. We grant 100% waivers to articles whose corresponding authors are based in Group A countries and 50% waivers to those who are based in Group B countries as classified by Research4Life (see: https://www.research4life.org/access/eligibility/ ). This section has its own submission website at https://mc.manuscriptcentral.com/e-videos .
